# Reactive Oxygen Species and the Lung Cancer Tumor Microenvironment: Emerging Therapeutic Opportunities

**DOI:** 10.3390/antiox14080964

**Published:** 2025-08-05

**Authors:** Kostas A. Papavassiliou, Amalia A. Sofianidi, Athanasios G. Papavassiliou

**Affiliations:** 1 First University Department of Respiratory Medicine, ‘Sotiria’ Chest Hospital, Medical School, National and Kapodistrian University of Athens, 11527 Athens, Greece; konpapav@med.uoa.gr; 2 Department of Biological Chemistry, Medical School, National and Kapodistrian University of Athens, 11527 Athens, Greece; amaliasof@med.uoa.gr

Lung cancer is the principal cause of cancer-related mortality globally, accounting for the high number of cancer-associated deaths amongst both men and women [1]. According to the American Cancer Society, the 5-year survival rate across all lung cancer stages is only 32% [2], implying that novel and more effective therapeutic approaches are required. Lately, the focus on cancer research has shifted towards targeting the metabolic reprogramming of malignant cells [3]. Indeed, the reprogramming of energy metabolism has long been recognized as a hallmark of cancer research, which confines distinct antioxidant capability in cancer cells [4]. Metabolic alterations distort reactive oxygen species (ROS) levels in the tumor microenvironment (TME) and ROS, in turn, impact cancer cell survival, progression, and response to treatment [5].

ROS comprise a divergent group of reactive entities that originate from molecular oxygen and include hydroxyl radicals (OH•), hydrogen peroxide (H_2_O_2_), superoxide radicals (O_2_^−^), and singlet oxygen (^1^O_2_). The main source of ROS generation in normal cells is cellular metabolism, primarily through oxidative phosphorylation in the mitochondria [5]. ROS are engaged in several biological processes such as inflammation, proliferation, and cell apoptosis [5]. Notably, malignant cells exhibit a significant increase in metabolic rate, leading to a heightened production of ROS. Low or moderate levels of ROS function as cancer promoters, activating malignant proliferation, invasion, and metastasis. On the contrary, high levels of ROS are toxic to tumor cells, triggering programmed cell death [6]. The unique characteristics of ROS in cancer cells present promising opportunities for exploitation in novel therapeutic strategies for lung cancer management.

Traditional therapeutic modalities have long used the generation of ROS to augment their anticancer effect. Cisplatin-based chemotherapy is one of the most common regimens employed in lung cancer treatment [7]. Cisplatin promotes and exploits the production of ROS, triggering cell death [8]. However, the dual role of ROS in cancer progression soon confers resistance of tumor cells to cisplatin [8]. Mitochondrial isocitrate dehydrogenase 2 (IDH2), an important NADP^+^-dependent enzyme involved in mitochondrial energy metabolism and redox homeostasis, was found to be upregulated in cisplatin-resistant lung cancer cells. Accordingly, it has been proposed that curbing IDH2 overexpression could help overcome cisplatin resistance [9]. Pharmacological ascorbate (ascorbic acid, vitamin C) has also been suggested as a complement to chemotherapy in non-small cell lung cancer (NSCLC). When this powerful antioxidant is present, iron metabolism is modified; redox active ferric iron (Fe^+3^), which exists in high levels in tumor cells, is reduced to ferrous iron (Fe^+2^) and as a result, ROS in the form of H_2_O_2_ is produced, impairing tumor cells [10]. A phase II clinical trial was conducted to investigate the antitumor outcomes of adding pharmacological ascorbate to platinum-based chemotherapy in patients with NSCLC. Treatment naïve advanced stage NSCLC patients received 75 g ascorbate twice per week intravenously in combination with carboplatin and paclitaxel every three weeks for four cycles. The response rates were numerically greater than the standard-of-care treatment options, even in patients with a low programmed death-ligand 1 (PD-L1) tumor proportion score (TPS) (<1%) who are less likely to benefit from the favorable effects of immunotherapy [11].

Natural compounds have been explored for their potential to augment oxidative stress in malignant cells. Bruceine D, a quassinoid extracted from the plant *Brucea javanica*, is a traditional Chinese medical compound that halts the proliferation of the A549 cell line (a human lung adenocarcinoma cell line) by inducing ROS overproduction and thus controlling apoptosis and autophagy via the mitogen-activated protein kinase (MAPK) signaling pathway [12]. Artesunate and dihydroartemisinin, derivatives of the natural compound artemisinin with established antimalarial properties, have also demonstrated antitumor potential. In NSCLC cell lines, they have been found to activate apoptosis and ferroptosis in a ROS-dependent manner [13]. Another natural compound that has exhibited anticancer effects through the induction of ROS-mediated apoptosis is thymoquinone, found in black cumin seeds of the plant *Nigella sativa*. In NSCLC cell lines, thymoquinone has the capacity to generate oxidative stress and increase ROS levels, which are detrimental to tumor cells [14]. Withangulatin A is another natural compound that is isolated from a popular medicinal and edible homologous plant, *Physalis angulata* L. It inhibits peroxiredoxin 6 (Prdx6), a ROS-scavenging enzyme, ultimately leading to ROS accumulation in NSCLC models in vitro [15]. Additionally, β-elemene, a sesquiterpene compound extracted from the medicinal herb *Curcuma wenyujin*, can be combined with erlotinib, halting glutathione (GSH; a ROS scavenging enzyme) and improving the sensitivity of NSCLC cells to erlotinib [16].

Several other therapeutic strategies have been tested during the past five years to exploit the deleterious effects of high ROS levels in lung tumor cells. IACS-010759 is a highly potent and selective small-molecule clinical inhibitor of mitochondrial complex I that impedes oxidative phosphorylation, leading to augmented ROS production and, eventually, cell death in *KRAS*-mutated NSCLC cells that have acquired resistance to the MAPK (MEK) inhibitor trabetinib [17]. Remarkably, the thioredoxin (Trx) system is a pivotal cellular antioxidant defense mechanism involved in regulating ROS. While crucial for maintaining the proper function of healthy cells, the Trx system pillars tumor growth by protecting tumor cells from excess oxidative stress. Auranofin, an orally administered gold salt, hinders the Trx system; recent in vitro and in vivo results demonstrate that auranofin can sensitize lung neuroendocrine tumor cells (NETs) and small cell lung cancer (SCLC) cells to sorafenib and hamper SCLC development, with a phase I/II clinical trial (NCT01737502) investigating the combination of auranofin and sorafenib in lung cancer [18]. The Trx system can also be targeted by shikonin, a natural naphthoquinone, and plumbagin, another natural naphthoquinone found in the roots, stems, and leaves of the genus *Plumbago*, especially in kelch-like ECH-associated protein 1 (KEAP1; a protein that acts as a negative modulator of the Nrf2 transcription factor)-mutant NSCLC cells, which comprise 20–30% of human lung adenocarcinomas [19,20].

In summary, ROS is a major component of the lung TME, both contributing to lung cancer progression and potentially serving as a therapeutic target. However, redox homeostasis is a very delicate process. ROS should be maintained at high levels to be toxic to malignant cells, as moderate levels are beneficial for cancer cell survival. Various preclinical data of agents targeting redox homeostasis, either as monotherapy or in combination with already established anticancer treatments, have been reported so far with promising results. What has yet to be determined is whether they will be effectively applied to the clinical setting.
